# Characterization of acute lung injury in the bleomycin rat model

**DOI:** 10.14814/phy2.15618

**Published:** 2023-03-10

**Authors:** Anil Hari Kadam, Jan E. Schnitzer

**Affiliations:** ^1^ Proteogenomics Research Institute for Systems Medicine (PRISM) La Jolla California USA

**Keywords:** acute lung injury (ALI), bleomycin, bronchoalveolar lavage fluid (BALF), contributing mediators/factors, detectable fibrogenesis, efficacy testing, experimental endpoints, key experimental features of ALI, mechanism of action, neutrophilic inflammation, progression of bleomycin‐induced ALI

## Abstract

The aim of this study was to describe and characterize the pathophysiological changes occurring during the early inflammatory phase (first 3 days) in the rat bleomycin model of lung injury preceding the development of fibrosis. Further, we wanted to understand the kinetics and factors contributing to bleomycin‐induced acute lung injury (ALI) and provide a robust, reliable and reproducible framework of features of ALI readouts to assess effects of therapeutics on bleomycin‐induced ALI in rats. We induced ALI in rats with intratracheal (i.t.) installation of bleomycin. The animals were sacrificed on predetermined time points, that is, Day 0, 1, 2, and 3 post the bleomycin challenge. We analyzed bronchoalveolar lavage fluid (BALF) and lung tissue to establish and assess relevant experimental features of ALI. We demonstrated that bleomycin induced key features of experimental ALI including a profound increase in neutrophils in BALF (50–60%), pulmonary edema, and lung pathology on Day 3 after challenge. Furthermore, we showed that TGF‐β1, IL‐1β, TNF‐α, IL‐6, CINC‐1, TIMP‐1, and WISP‐1 were induced by studying their kinetic profile during the first 3 days after bleomycin injury consistent with their known role ALI. We also confirmed that detectable fibrogenesis occurs at the earliest on Day 3 after injury based on collagen content, along with changes in the TGF‐β/Smad signaling pathway and increased expression of Galectin‐3, Vimentin, and Fibronectin in lung homogenate. Our report presents robust features and contributing mediators/factors to the pathology of bleomycin‐induced ALI in rats on Day 3. The kinetic data provide insights on the progression of ALI and a detailed understanding of early events before actual fibrosis development. This set of experimental endpoints is very appropriate and invaluable for efficacy testing of potential novel therapeutic treatments (single or combined) in ALI and understanding their mechanism of action.

## Graphical abstract

We demonstrate that bleomycin induces key features of experimental ALI including a profound increase in neutrophils in BALF (50–60%), pulmonary edema, and lung pathology on Day 3 after the challenge. Furthermore, we showed that key inflammatory markers including TGF‐β1, IL‐1β, TNF‐α, IL‐6, CINC‐1, TIMP‐1, and WISP‐1 were induced by studying their kinetic profile during the first 3 days after bleomycin injury. Our work adds to the knowledge of current ALI models and provides a set of experimental endpoints appropriate for efficacy testing of potential novel therapeutic treatments in ALI and for understanding mechanisms of action of therapeutics under investigation.

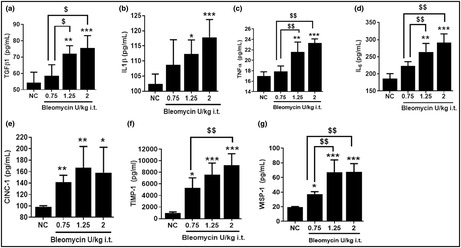

## 1 INTRODUCTION

Acute lung injury (ALI) is a clinical disease marked by respiratory failure due to the disruption of the epithelial and endothelial barrier, flooding of the alveolar compartment with protein‐rich fluid, and recruitment of neutrophils into alveolar space (Manicone, 2009; Zhao et al., 2021). Research regarding the molecular pathophysiology of ALI is ongoing, with the aim to develop prognostic molecular biomarkers and molecular‐based therapy (Butt et al., 2016). Animal models have contributed significantly to our understanding of the pathogenesis and pathophysiology of ALI and acute respiratory distress syndrome (ARDS). Investigators have used a variety of small and large animal models to understand the mechanisms of injury to both the lung endothelial and epithelial barriers, as well as to test novel therapeutic strategies (Matthay & Howard, 2012). Accurate and reproducible assessment of experimental lung injury and inflammation is critical for basic and translational research.

Bleomycin, a chemotherapeutic drug for human malignant tumors (Zhao et al., 2021) induces progressive inflammation and subsequently fibrosis in rodents (Tashiro et al., 2017). Early inflammation comprises neutrophil infiltration with increased Th1 T helper type immune response in the lung (Peng et al., 2013). Bleomycin produces damage/necrosis of the alveolar epithelium, capillary congestion, and perivascular permeability leading to edema and the formation of hyaline membranes, which are all phenotypes of ALI. Moreover, persistent injury leads to the development of pulmonary fibrosis with excessive production and deposition of collagen in the lung (Foskett et al., 2014). The bleomycin model is well characterized, extensively used, provides invaluable insights into the pathophysiology of pulmonary fibrosis, and allows testing of novel therapeutics. However, the early inflammatory stages have not been extensively studied in rats. The effect of bleomycin dose on disease progression in the early inflammatory phase of ALI (0–3 days) needs to be systematically evaluated. Therefore, in the current study we characterize the utility of a 3‐day bleomycin rat model for investigating ALI pathophysiology. We comprehensively identify experimental conditions that best reflect commonly accepted features of ALI disease, describe mechanisms of ALI progression, and identify earliest time points of detectable fibrogenesis in rats. We also provide a framework of robust and reproducible readouts which allows the assessment of ALI, that could be used to quantify the effects of therapeutic interventions and possible disease pathways in the future.

## 2 MATERIALS AND METHODS

### 2.1 Animals

Female Sprague Dawley rats (ENVIGO, USA) weighing 200–220 g were housed at 25°C with a 12 h day/night cycle, and food and water ad libitum. Animals were maintained under these conditions until the end of the study. Care and experiments were approved by the PRISM Institutional Animal Care and Use Committee.

### 2.2 Induction of acute lung injury and selected times for endpoint analysis

On Day 0, rats were anesthetized with inhaled isoflurane (3%–5%) and received a single instillation it of bleomycin (0.75, 1.25 or 2 U/kg) using a 18 gauge needle attached to a 1 mL tuberculin syringe. Control animals received 300 μL 1X PBS. After instillation, rats were allowed to recover from anesthesia, kept warm, and returned to their cages with free access to food and water. Animals were sacrificed on Day 0, 1, 2 or 3, and ALI relevant endpoints were analyzed.

### 2.3 Bronchoalveolar lavage (BAL)

Three days after bleomycin instillation, animals were euthanized with Euthasol (100–120 mg/kg) by intraperitoneal (ip) injection. The trachea was exposed following a small incision to the skin, and BAL was performed 3 times using a plastic cannula with 2 mL 1X PBS (pH = 7.4). Volumes of individual BAL aspirates were pooled.

### 2.4 Assessment of pulmonary inflammatory cells

We mixed equal volumes of BAL fluid (BALF) and Turk's solution and counted total leukocytes manually using a hemacytometer (Hausser Scientific). The remaining fluid was centrifuged at 4000 RPM for 5 min at 4°C; aliquots of BALF supernatant were collected aseptically and stored at −80°C until analysis. Cell pellets were reconstituted in rat serum and stained with Leishman solution on frosted glass slides (Leica Biosystems). Using a light microscope (BX2, Olympus, Tokyo, Japan) at 100X magnification, 500 cells/slide were counted. Cells were categorized based upon morphology into neutrophils, lymphocytes, eosinophils, or macrophages.

### 2.5 Lung harvest for protein and collagen determination as well as histology

After BAL, right lungs were harvested from animals for biochemical assays, washed in 1X PBS, placed in 1 mL PBS containing 0.1% (v/v) protease and phosphatase inhibitor cocktail, and stored at −80°C until use. For histology, left lungs were carefully removed and stored in 10% neutral buffered formalin (NBF).

### 2.6 Assessment of pulmonary edema

Pulmonary edema was assessed by measuring BALF protein content, whole lung weight, lung index, and percentage of water content in whole lung. BALF protein concentration was determined by colorimetric detection by bicinchoninic acid (BCA) assay. After BAL, in one set of animals, lungs were harvested, washed in 1X PBS to remove debris, blotted using tissue paper, and weighed (wet weight). Lung index was determined by dividing lung weight with body weight. In a separate set of rats, lungs were harvested without performing BAL to determine the severity of pulmonary edema using the ratio of whole wet to dry weight. To obtain dry weight, lungs were incubated in an oven (45°C for 24 h). The percentage of water content was determined by the ratio of wet and dry lung weight.

### 2.7 Assessment of lung collagen content by Sircol soluble collagen assay

Fibrosis was assessed by quantifying total soluble collagen using the *Sircol collagen assay kit* (Biocolor Ltd.). Briefly, wet right lungs were washed in 1X PBS and homogenized in 5 mL of CHAPS detergent buffer. The lung homogenate was mixed with equal volume of acid pepsin solution (5 mg/mL of 0.5 M acetic acid) and incubated over night at 4°C. Following centrifugation, the supernatant was assayed for soluble collagen content according to the manufacturer's instructions. Absorbance at 555 nM was read on *VersaMax ELISA Microplate Reader* (Molecular Devices, LLC.). Lung collagen data were expressed as μg of soluble collagen per right lung of rat.

### 2.8 Quantification of biomarkers of inflammation in rat lungs

Inflammatory cytokine/chemokine levels in BALF harvested from bleomycin challenged rats were analyzed using commercially available ELISA assays kits according to manufacturer's instructions. The rat Quantikine ELISA kit for TGF‐β1, IL‐1β, TNF‐α, IL‐6, TIMP‐1, CINC‐1, and WISP‐1 was purchased from R&D System, USA. The level of each biomarker is expressed in (pg/mL) of BALF.

### 2.9 Lung preparation and western blot analysis

Lungs were homogenized on ice in 5 mL CHAPS buffer containing protease and phosphatase inhibitors using a homogenizer (PT3100‐POLYTRON, Kinematica, NY). Homogenates were centrifuged at 27,670 *g* for 15 min at 4°C, and the supernatant was stored at −80°C until use. Total protein concentrations were determined using BCA assay. Proteins (50 μg) were separated on Novex 4–12% Tris‐Glycine gels, transferred onto nitrocellulose membranes, blocked with SuperBlock™ for 2 h at room temperature (RT) and incubated with primary antibodies diluted in TRIS buffered saline pH = 7.4 (TBS) containing 0.1% Tween‐20 following manufacturer's instructions. After washing with TBS‐Tween, membranes were incubated with goat anti‐rabbit IgG HRP‐linked secondary antibody (1:10,000) for 1 h at RT and proteins were visualized by enhanced chemiluminescence. Signals were quantified using ImageJ software.

### 2.10 Lung histology and injury scoring

For histology, left lungs were processed using a routine histology protocol. Paraffin‐embedded tissue (4 μm slides) was stained with hematoxylin and eosin. Pathological changes in lung tissue were assessed using the following criteria adapted from previously published protocols (Anil et al., 2022): 1. cell infiltration severity 0–4 (0: normal, 1: mild, 2: moderate, 3: severe, 4: highly severe), 2. presence and absence of hyaline membranes (0: absence and 1: presence), 3. presence and absence of pulmonary edema (0: absence and 1: presence), 4. alveolar septal thickening 0–3 (0: Normal, 1: 2X, 2: 3X, 3: >3x) in total 20 random microscopic fields (20X magnification). Lung injury scores reflect the sum of criteria above.

### 2.11 Statistical analysis

All data are presented as the mean ± standard deviation (SD). The data were analyzed using one‐way ANOVA followed by Dunnett's test for multiple comparisons or unpaired t‐test as needed using GraphPad prism. A *p* value <0.05 compared with normal control or Day 0 was set as statistically significant.

## 3 RESULTS

### 3.1 Bleomycin concentrations and ALI phenotype on Day 3 after exposure in rats

The American Thoracic Society has identified features in rodent models of ALI that are widely accepted as relevant readouts for exploring human ALI (Matute‐Bello et al., 2011). However, comprehensive data during the early inflammatory phase in these models are incomplete. Therefore, to systematically characterize the effects of bleomycin dose (0.75 to 2 U/kg) on ALI disease phenotype, we quantified neutrophilic lung inflammation, pulmonary edema formation, and changes in biochemical markers of lung injury in a 3‐day rat model.

**FIGURE 1:**
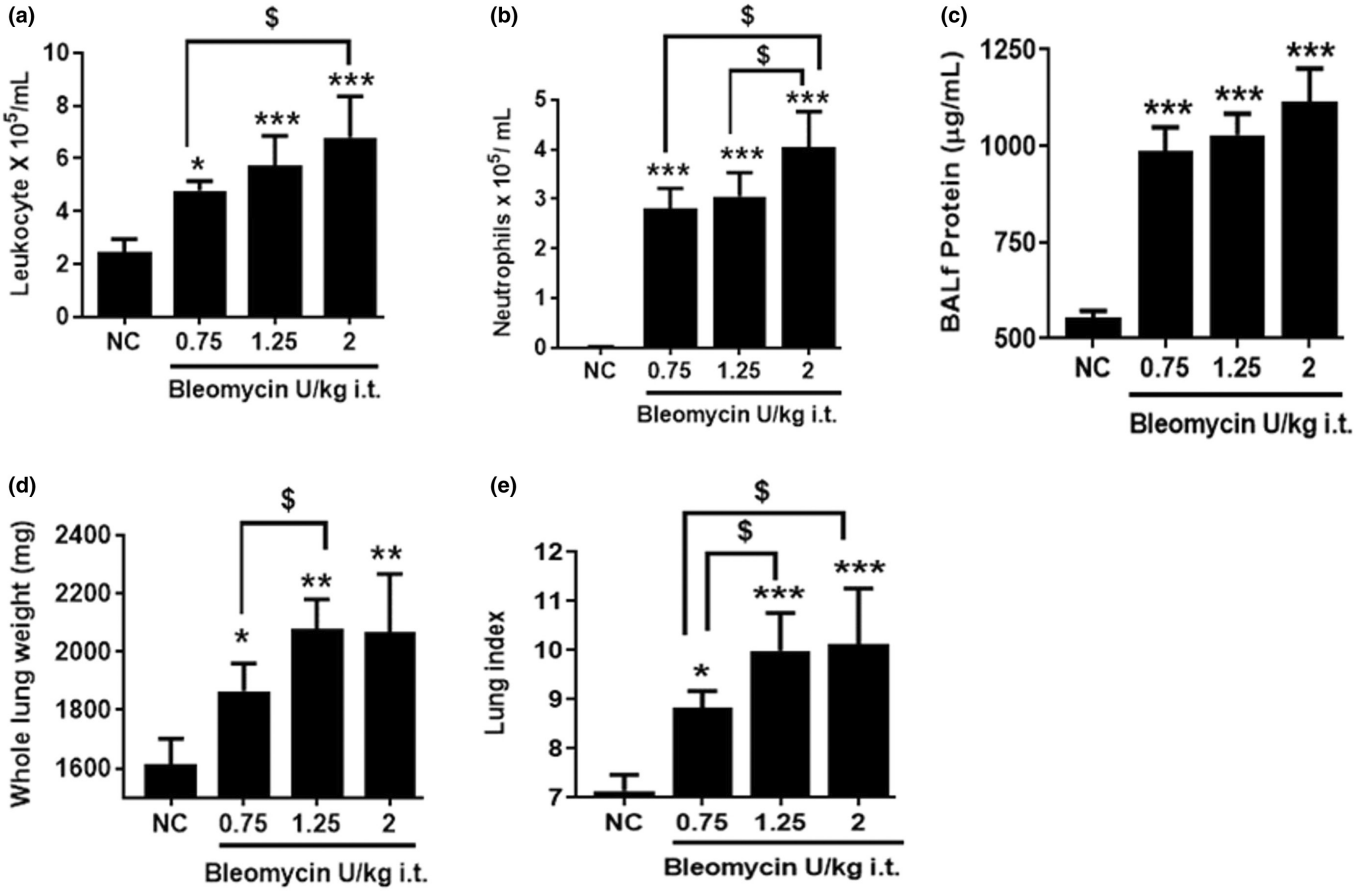
Effect of i.t. bleomycin concentrations on infiltration of inflammatory cells in BALF and pulmonary edema on Day 3. (a) Leukocytes, (b) Neutrophils, (c) Protein content, (d) Whole lung weight, (e) Lung index. Data are expressed as mean ± SD of *n* = 4–5 rats/group. *p < 0.05; **p < 0.01; and ***p < 0.001 vs *NC. $p <* 0.05 by unpaired t‐test.

First, we assessed the inflammatory response in rats challenged with it administration of 0.75, 1.25, and 2 U/kg bleomycin at Day 3. Gross inspection of the lungs did not indicate hemorrhage. In addition, please note that we administered bleomycin intratracheally instead of intravenously at fairly low doses to minimize vascular damage, consistent with past reports (Matute‐Bello et al., 2011). Bronchoalveolar lavage fluid (BALF) was harvested, and the inflammatory response was assessed by performing differential cell counts. We observed a significant (*p <* 0.05*, p <* 0.001), dose‐dependent increase in leukocyte recruitment into the bronchoalveolar space in rats treated with 0.75 to 2 U/kg bleomycin compared to normal controls (NC) (Figure 1a). The increase in total neutrophils and lymphocytes was also significant (*p <* 0.001, *p <* 0.05) in BALF obtained from 0.75, 1.25, and 2 U/kg bleomycin treated rats (Figure 1b, and Figure S1). Inflammatory cells were comprised of mostly neutrophils (50–60%) with negligible amounts of lymphocytes (4–6%) and eosinophils (5–7%). In contrast, BALF from NC was comprised mostly of macrophages (97–100%) (Figure S2). Leukocytes and neutrophils increased significantly between 0.75 U/kg and 2 U/kg bleomycin exposure, whereas between 1.25 U/kg and 2 U/kg a statistical increase was only seen in neutrophils (*p <* 0.05) but not leukocytes. Lymphocytes levels remained constant when 0.75–2 U/kg were used. No statistical difference between 0.75 and 1.25 U/kg bleomycin on leukocyte, neutrophil, and lymphocyte recruitment was detected (Figure 1a,b, and Figure S1). The analysis of inflammatory cells in BALF revealed that bleomycin at all tested doses resulted in significant pulmonary neutrophilia on Day 3, a hallmark of ALI.

We then examined changes in BALF protein content, whole lung weight and lung index to assess pulmonary edema. Significantly (*p <* 0.001) elevated BALF protein content (Figure 1c), increased lung weight, and lung index readouts (Figure 1d,e) were observed in rats challenged with 0.75, 1.25, and 2 U/kg bleomycin compared to NC. Using 1.25 U/kg bleomycin compared to 0.75 U/kg significantly (*p <* 0.05*)* increased lung weight and lung index. With 2 U/kg bleomycin, the lung index increased significantly (*p <* 0.05*)* compared to 0.75 U/kg, whereas lung weight and BAL protein content did not (Figure 1c–e). The increase in BALF protein content, lung weight, and lung index measures indicate robust pulmonary edema formation 3 days after rats were subjected to 0.75–2 U/kg bleomycin.

**FIGURE 2:**
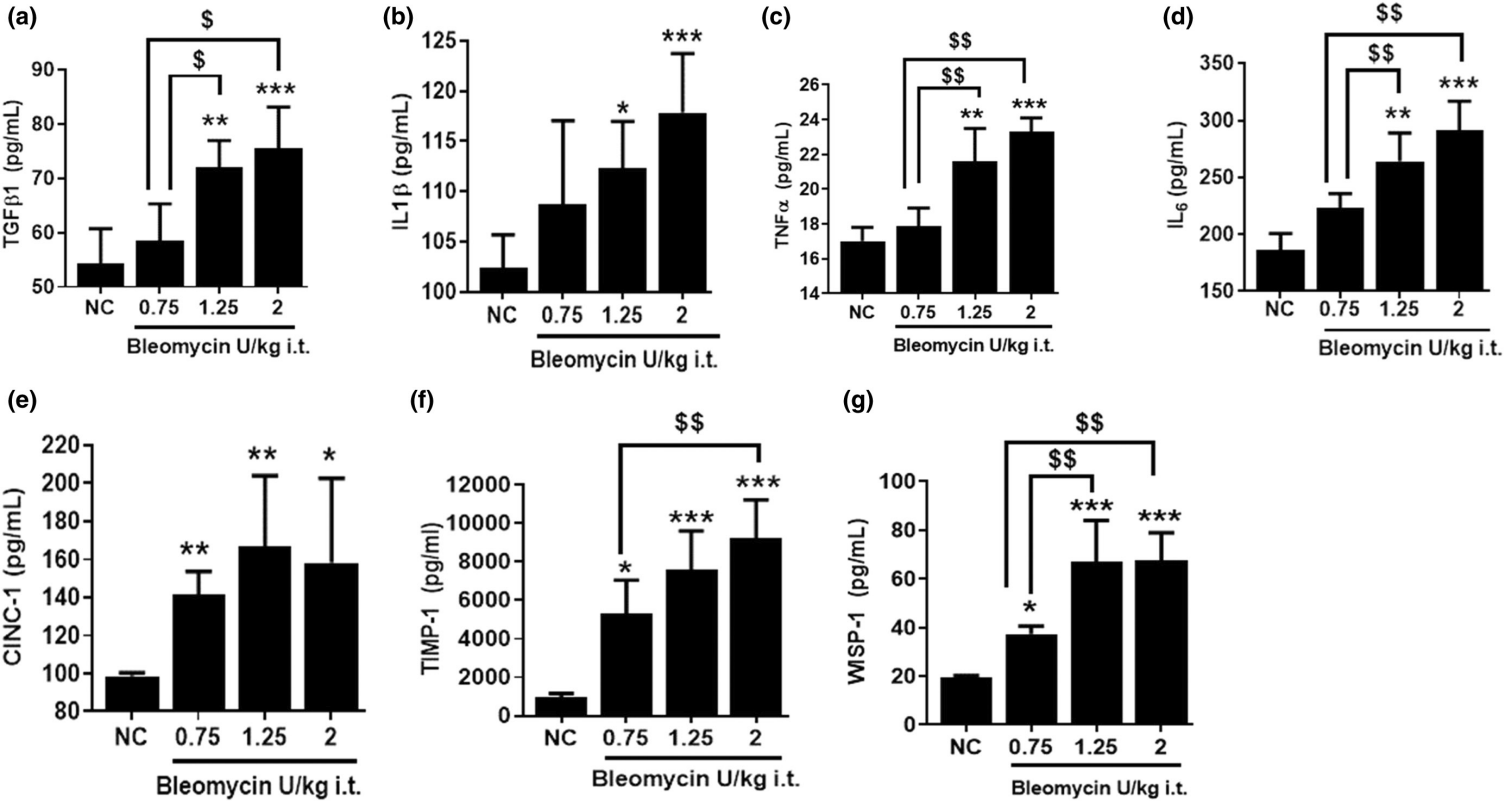
Effect of i.t. bleomycin concentrations on BALF inflammatory meditators on Day 3. (a) TGF‐β1, (b) IL‐1β, (c) TNF‐α, (d) IL‐6, (e) CINC‐1, (f) TIMP‐1, and (g) WISP‐1. Data are expressed as mean ± SD of *n* = 4–5 rats/group. *p < 0.05; **p < 0.01; and ***p < 0.001 vs *NC. $p <* 0.05; *$$p <* 0.01 by unpaired t‐test.

To further elucidate dose effects of bleomycin on various biochemical markers of inflammatory lung injury, we quantified TGF‐β1, IL‐1β, TNF‐α, IL‐6, TIMP‐1, CINC‐1, and WISP‐1 concentrations in BALF on Day 3 post challenge. All biomarkers were significantly (*p <* 0.05*, p <* 0.01) upregulated following the 1.25 and 2 U/kg bleomycin challenge compared to NC (Figure 2a–g). In contrast, 0.75 U/kg bleomycin resulted in no significant changes of TGF‐β1, IL‐1β, TNF‐α, and IL‐6 levels, but TIMP‐1, CINC‐1, and WISP‐1 levels were significantly (*p <* 0.05) elevated compared to NC (Figure 2a–g). When we compared 1.25 U/kg to 0.75 U/kg bleomycin (Figure 2a–g), the concentration of BALF TGF‐β1, TNF‐α, IL‐6, and WISP‐1 was significantly increased (*p <* 0.05*, p <* 0.01) whereas levels of IL‐1β, CINC‐1, and TIMP‐1 were higher but not significantly. Similarly, 2 U/kg bleomycin induced a significant (*p <* 0.01) local release of BALF TGF‐β1, TNF‐α, IL‐6, TIMP‐1, and WISP‐1, whereas IL‐1β and CINC‐1 levels were higher, but not significantly compared to 0.75 U/kg. Both 1.25 and 2 U/kg showed approximately similar levels of increased inflammatory biomarkers due to bleomycin (Figure 2a–g). Our data demonstrate that several biochemical mediators of tissue inflammation were elevated on Day 3 in rats challenged with bleomycin.

### 3.2 Kinetic of experimental features of ALI induced by bleomycin in rats

We observed a robust induction of ALI in rats with 2 U/kg. We chose this dose to study the kinetic of ALI using our established readouts (see above) to understand how ALI progresses from the day of induction to Day 3. As the rodent bleomycin model is the classical model for lung fibrosis research, we were also keen to elucidate when collagen production was initiated after the bleomycin challenge.

**FIGURE 3:**
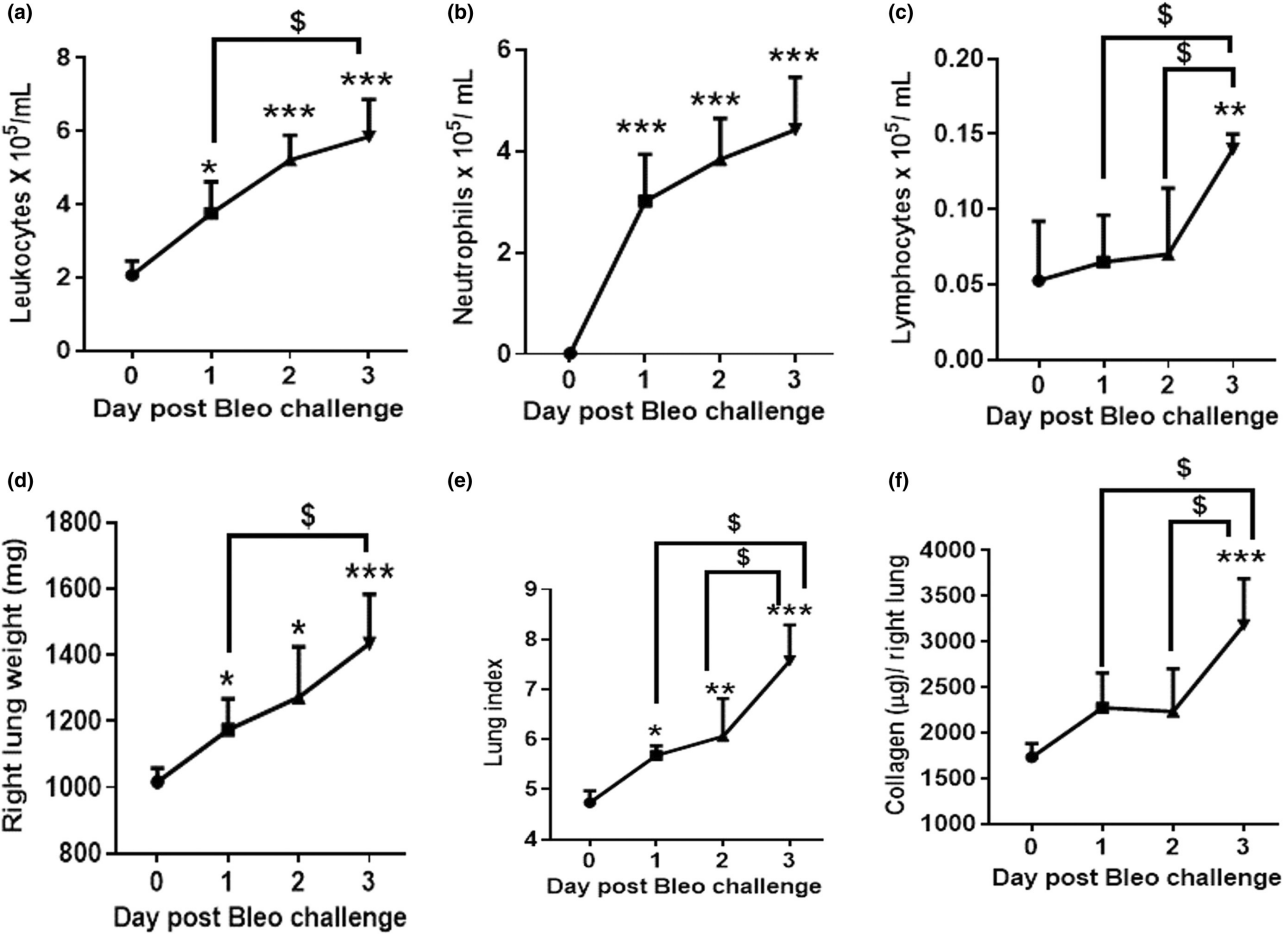
Effect of bleomycin i.t. challenge on the kinetic of pulmonary inflammatory cell infiltration and lung parameters. (a) Leukocytes, (b) Neutrophils, (c) Lymphocytes, (d) Right lung weight, (e) Lung index, and (f) Lung collagen content. Data are expressed as mean ± SD of *n* = 5 rats/group. *p < 0.05; **p < 0.01; and ***p < 0.001 vs Day 0. $*p* < 0.05 by unpaired t‐test.

To document the kinetic of pulmonary cell infiltration, animals were challenged with 2 U/kg bleomycin and differential cell counts in BALF were determined as described above. Leukocytes and neutrophils increased significantly during Days 1 to 3 (*p <* 0.05*, p <* 0.001) whereas lymphocytes only increased on Day 3 (*p <* 0.05*, p <* 0.01) compared to Day 0. Macrophages significantly decreased on Day 1 but recovered on Days 2 and 3 after the bleomycin challenge compared to Day 0 (Figure 3a–c and Figure S3). The increase in leukocytes and neutrophils from Day 0 to Day 3 was time dependent where leukocyte counts significantly (*p <* 0.05*)* increased on Day 3 compared to Day 1. Similarly, the lymphocyte increase was significantly higher on Day 3 compared to Days 1 and 2 (Figure 3c). Leukocyte and neutrophil counts were higher on Day 3 compared to Day 2 but not statistically different. We showed that neutrophilic cellular infiltration in the lung progressed from the day of induction to Day 3.

We then characterized the kinetic of pulmonary edema and fibrogenesis in rats challenged with 2 U/kg bleomycin. Lung weight and lung index increased significantly during Days 1 to 3 (*p <* 0.05, *p <* 0.01) whereas lung collagen content significantly (*p <* 0.01) increased only on Day 3 compared to Day 1 (Figure 3d–f). The increase in lung weight was greater on Day 2 compared to Day 1 but not significantly. On Day 3, however, lung weight was significantly (*p <* 0.05) higher compared to Day 1. Also, the increase in the lung index was significant (*p <* 0.05*)* on Day 3 compared to Days 1 and 2. Most importantly, lung collagen content significantly (*p <* 0.05) increased on Day 3 compared to Days 1 and 2 (Figure 3d–f). In our model, we detected signs of fibrogenesis (collagen increase) as early as 3 days after the bleomycin challenge following progressive edema formation.

**FIGURE 4:**
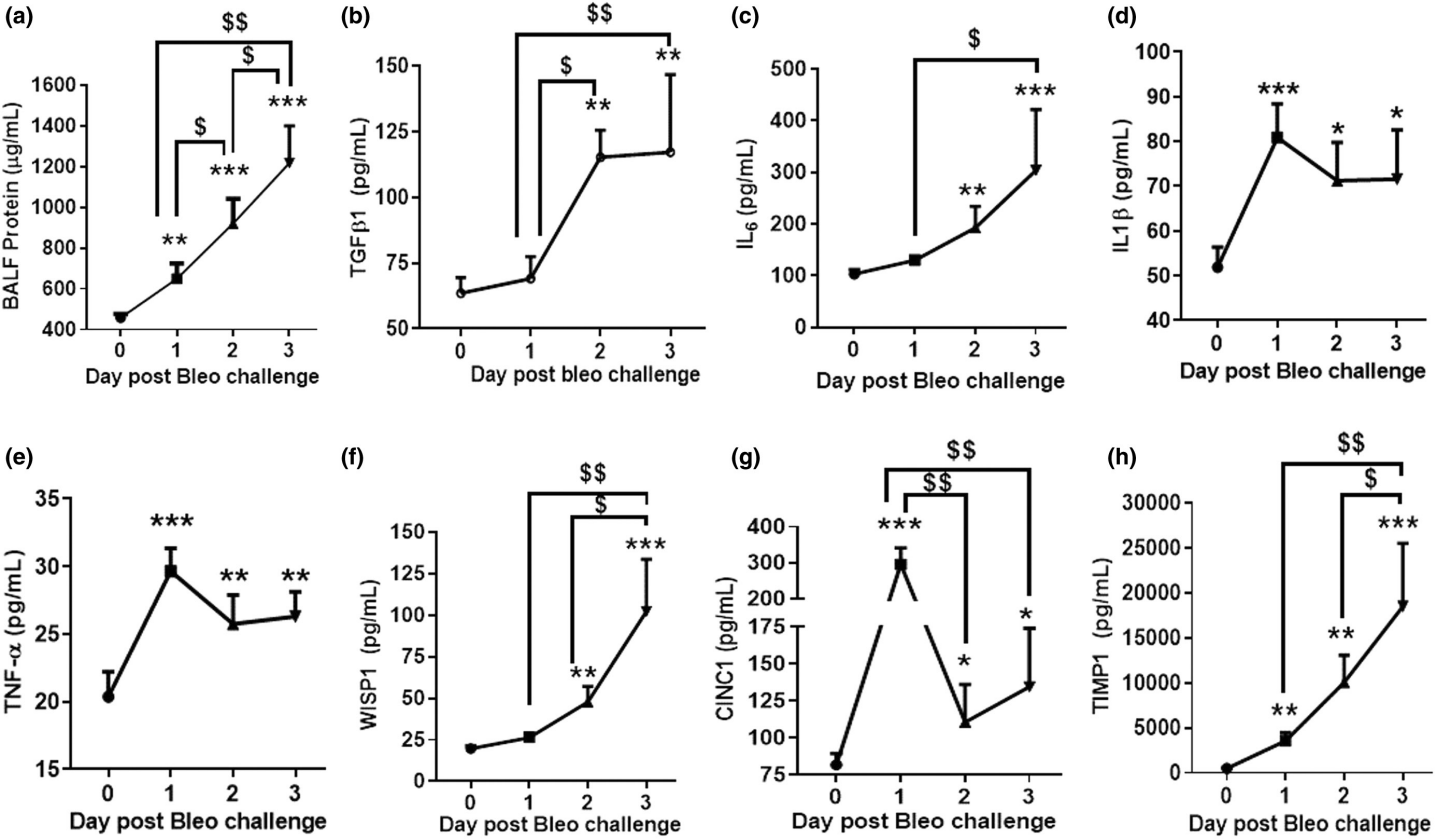
Effect of bleomycin i.t. challenge on the kinetic of BALF inflammatory biomarkers. (a) Protein content, (b) TGF‐β1, (c) IL‐6, (d) IL‐1β, (e) TNF‐α, (f) WISP‐1, (g) CINC‐1, and (h) TIMP‐1. Data are expressed as mean ± SD of *n* = 5 rats/group. *p < 0.05; **p < 0.01; and ***p < 0.001 vs Day 0. $*p* < 0.05; $$*p* < 0.01 by unpaired t‐test.

To better understand the contribution of various inflammatory mediators in the progression of bleomycin‐induced lung injury, we measured protein content, TGF‐β1, IL‐6, IL‐1β, TNF‐α, WISP‐1, CINC‐1, and TIMP‐1 in BALF during Days 1 to 3 post bleomycin. The levels of BALF IL‐1β, TNF‐α, and CINC‐1 significantly (*p <* 0.05*, p <* 0.01) increased on Day 1 whereas on Days 2 and 3 a significant (*p <* 0.05) increase of all mediators was observed (Figure 4a–h). The levels of BALF protein content, WISP‐1, TIMP‐1 rose post the bleomycin challenge whereas the levels of BALF IL‐1β, TNF‐α, and CINC‐1 declined on Days 2 and 3 compared to Day 1. In particular, levels of IL‐1β and TNF‐α on Days 2 and 3 were the same compared to Day 1; however, CINC‐1 levels significantly *(p <* 0.01*)* decreased on Days 2 and 3 compared to Day 1. The levels of TGF‐β1 on Days 2 and 3 were similar but significantly higher than on Day 1. Levels of protein content, IL‐6, WISP‐1, and TIMP‐1 on Day 3 were significantly higher than on Day 1. Also, the levels of WISP‐1 and TIMP‐1 significantly increased on Day 3 compared to Day 2. Our data indicate that bleomycin triggered the release of inflammatory mediators starting on Day 1 continuing up to Day 3 (Figure 4a–h).

### 3.3 Bleomycin induces robust and reproducible features of ALI and initiates fibrogenesis on Day 3

After determining the effects of bleomycin dose and time on ALI development, we were keen to provide a robust, reliable, and reproducible framework of ALI readouts that can be used to assess disease phenotype in vivo to determine the effects of therapeutic interventions and understand mechanisms of action. Therefore, we increased the numbers of animals up to 20–25 to establish reliable and robust readouts. We also expanded our investigation and assessed pulmonary edema without performing BAL, molecular signaling pathway components (P‐Smad2/3), Galectin‐3 (molecular target for inflammation and fibrosis), activation of EMT which is clinically relevant for fibrogenesis, Vimentin, and Fibronectin, an extracellular matrix component present during the fibroproliferative phase of inflammation. Additionally, we performed histopathology on Day 3.

**FIGURE 5:**
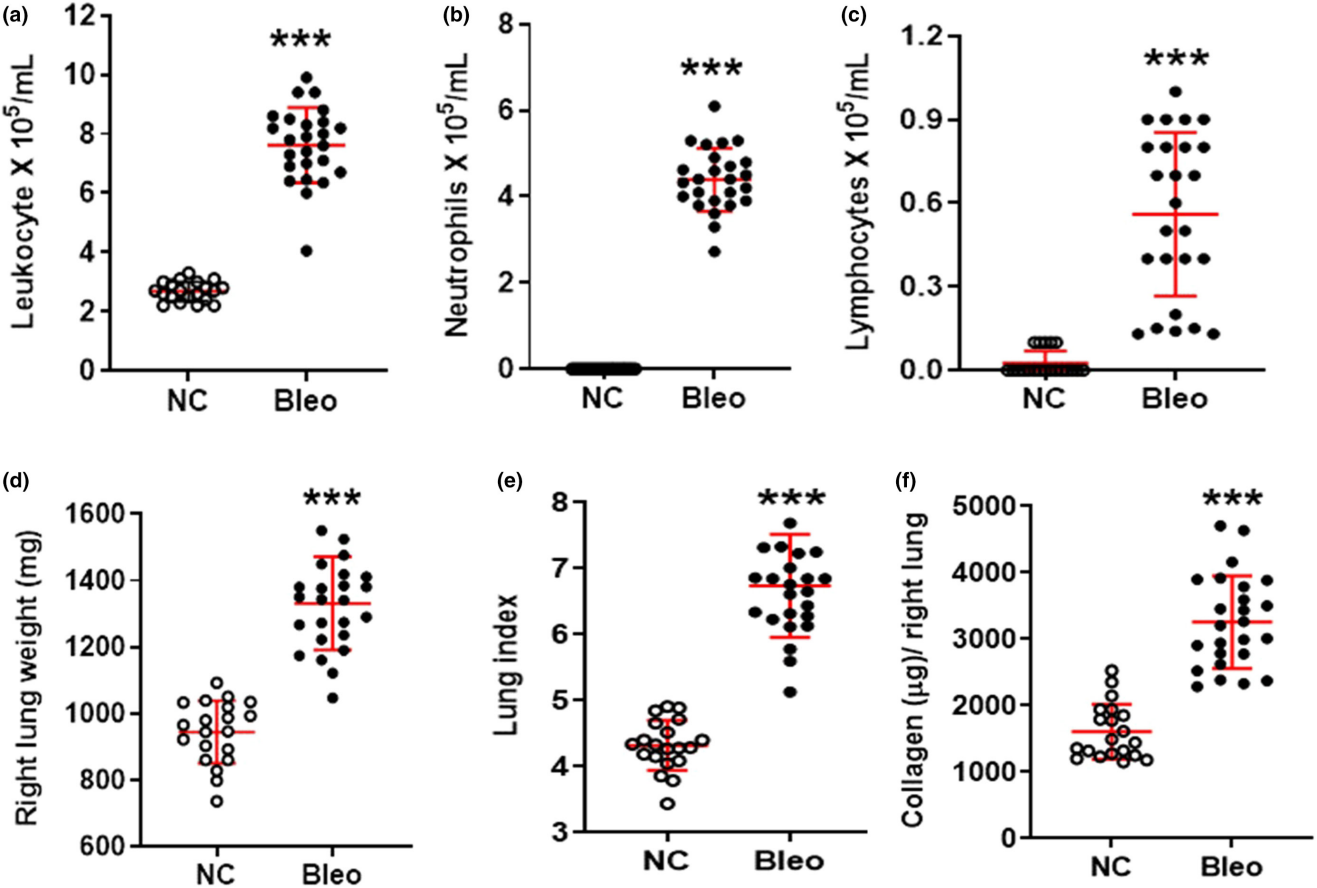
Effect of bleomycin i.t. challenge on pulmonary inflammatory cell infiltration and lung parameters on Day 3. (a) Leukocytes, (b) Neutrophils, (c) Lymphocytes, (d) Right lung weight, (e) Lung index, and (f) Lung collagen content. Data are expressed as mean ± SD of *n* = 20–25 rats/group. ***p < 0.001 vs *NC*.

**FIGURE 6:**
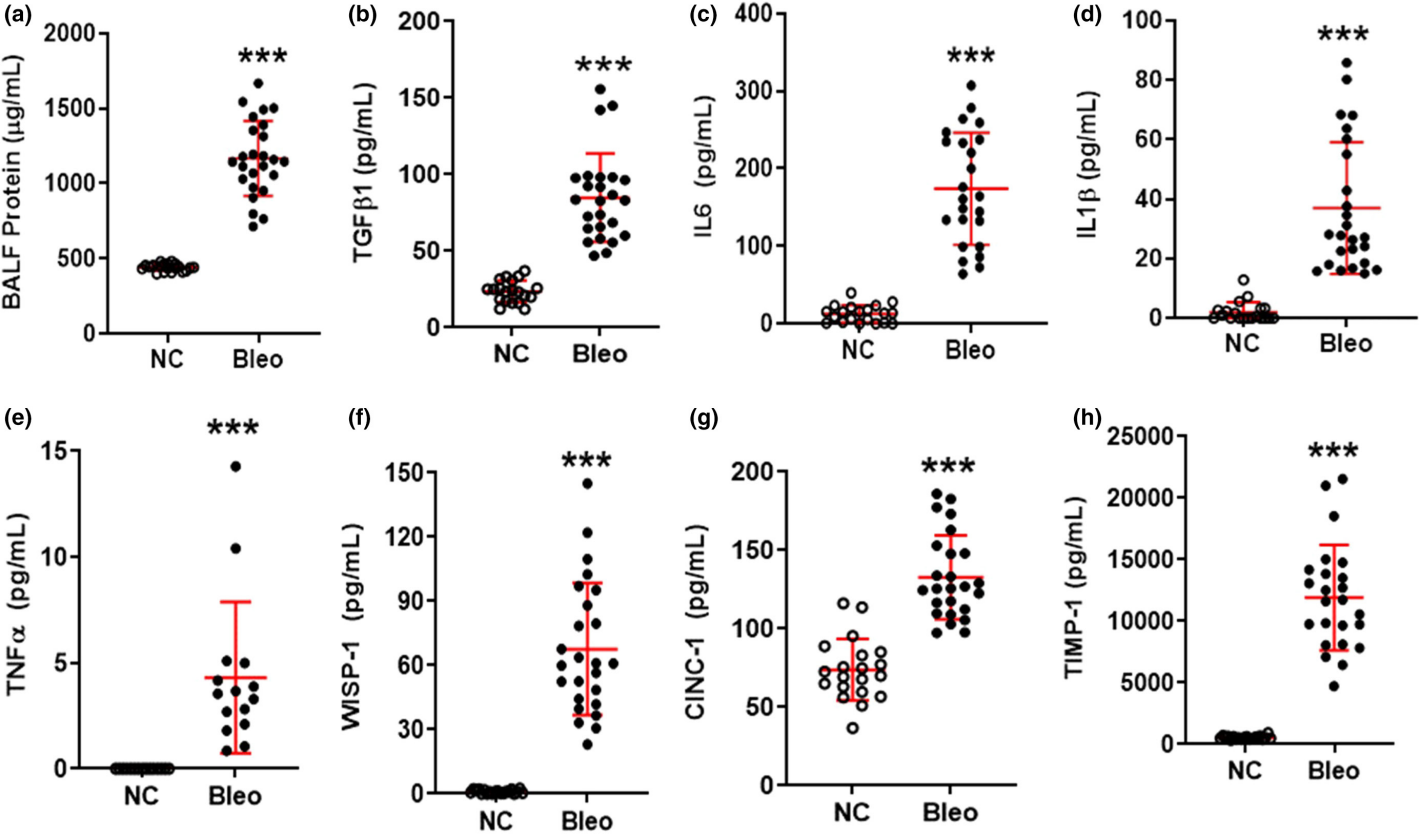
Effect of bleomycin i.t. challenge on BALF inflammatory biomarkers on Day 3. (a) Protein content, (b) TGF‐β1, (c) IL‐6, (d) IL‐1β, (e) TNF‐α, (f) WISP‐1, (g) CINC‐1, and (h) TIMP‐1. Data are expressed as mean ± SD of *n* = 20–25 rats/group, *TNF‐α *n* = 15 rats/group. ***p < 0.001 vs *NC*.

**FIGURE 7:**
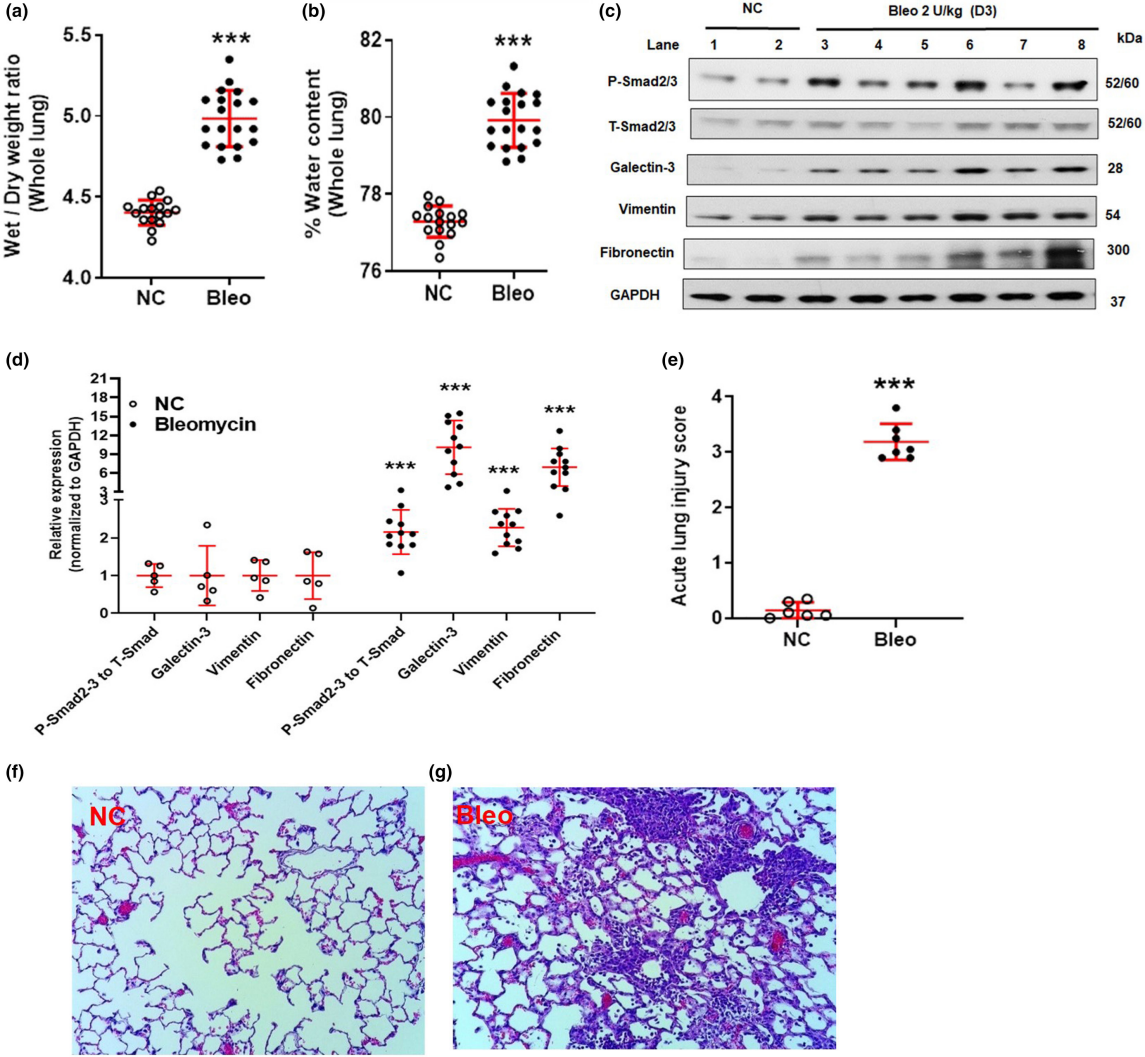
Effect of bleomycin i.t challenge on pulmonary edema, TGF‐β‐Smad pathway, molecular target, EMT marker, and ECM component, lung histopathology and ALI score on Day 3. Pulmonary edema: (a) Whole lung wet to dry weight ratio at 24 h, (b) % Whole lung water content at 24 h. Data are expressed as mean ± SD for *n* = 16–19 rats/group. Protein expression: c and d Protein expression of P‐Smad2/3, Galectin‐3, Vimentin, and Fibronectin, *n =* 5–11/group. Histology: (e) ALI score, (f) Normal lung, (g) Bleomycin treated lung, *n* = 6‐7/group. ***p < 0.001 vs *NC*.

When we assessed the inflammatory response in BALF of rats challenged with 2 U/kg bleomycin at Day 3 after exposure, we saw a significant increase of leukocytes, neutrophils, and lymphocytes (*p <* 0.001*)* but not macrophages (Figure 5a–c and Figure S3). Similarly, 2 U/kg of bleomycin induced a significant and robust (*p <* 0.001*)* increase in inflammatory mediators such as TGF‐β1, IL‐6, IL‐1β, TNF‐α, WISP‐1, CINC‐1, and TIMP‐1 (Figure 6b–h). When quantifying the effects on lung collagen content by Sircol assay, we saw that lung collagen content increased significantly compared to NC (*p <* 0.001*)* (Figure 5f). Additionally, the bleomycin challenge resulted in significant (*p <* 0.001*)* increase of protein content (Figure 6a). We assessed edema formation with and without performing BAL. With BAL, bleomycin treatment caused significant (*p <* 0.001) increase in lung weight and lung index *(*Figure 5d,e) and the same result was obtained without BAL, when we harvested lung, recorded wet weight, and dried it until constant weight which was achieved after 24 h (Figure S4). Some of this increase in lung weight could come from vasodilation and increase in blood volume in the lung. We observed a significant (*p <* 0.001*)* increase in the wet to dry weight ratio and a significant (*p <* 0.001*)* increase in the percentage of water content on Day 3 compared to NC (Figure 7a,b and Figure S4). We then focused on changes in lung tissue and detected the expression of phospho‐Smad2/3, Galectin‐3, Vimentin, and Fibronectin using Western blot analysis. Bleomycin significantly (*p <* 0.01*, p <* 0.001*)* increased phospho‐Smad2/3, Galectin‐3, Vimentin, and Fibronectin protein expression compared to NC (Figure 7c,d). Concomitantly, we detected pathological changes in the lung using H&E staining. As shown in Figure 7, lung tissues from the control group revealed a normal structure and no histopathological changes (Figure 7f). In contrast, lung tissues from the bleomycin group were markedly damaged; these tissues had inflammatory cell infiltration, interstitial edema, thickness of alveolar septa (Figure 7g) as evidenced by increases in lung injury scores (Figure 7e).

Our 3‐day rat model data show complete, robust and reproducible experimental features of ALI and early initiation of fibrogenesis after bleomycin challenge.

## 4 DISCUSSION

Neutrophil recruitment is a hallmark of ALI (Abraham, 2003). In human and animal studies, neutrophil numbers in BALF correlate with ALI severity and are predictive of mortality (Grudzinska & Sapey, 2018). In rodents, bleomycin first leads to the recruitment of neutrophils followed by the development of fibrosis (Herrmann et al., 2017). Our studies confirmed these results and showed that 2 U/kg induced robust progressive neutrophil infiltration beginning on Day 1 and lasting until Day 3.

Pulmonary edema, another feature of ALI, results from neutrophil‐induced damage of the alveolar capillary interface and increases the leakiness of the epithelial barrier (Herrero et al., 2018), thus contributing to alveolar flooding and disruption of alveolar fluid clearance (Geiser et al., 2001). In BALF from patients with ALI and ARDS, products secreted by activated neutrophils such as ROS, proteinases, and soluble inflammatory mediators are elevated and implicated in alveolar epithelium damage and pulmonary edema (Pittet et al., 1997). In animal models, increased lung weight/index, lung wet to dry ratio, and BALF protein content are used as indicators of edema (Kenyon et al., 2002). Here, we showed that bleomycin at 0.75–2 U/kg increased protein content to a similar degree. Also, lung weight and lung index parameters were significantly increased due to bleomycin 1.25 to 2 U/kg >0.75 U/kg. Additionally, lung weight and lung index increased from Day 1 to Day 3 post bleomycin challenge (2 U/kg) indicating progressive pulmonary edema formation. An independent assessment of pulmonary edema without performing BAL also showed significant induction of pulmonary edema due to bleomycin on Day 3 based on increased lung wet to dry weight ratio and percentage water content.

Cytokines play a critical role in inflammatory responses underlying fibrosis induction in injured tissues (Borthwick et al., 2013). Pulmonary inflammation in ALI is in part mediated by proinflammatory cytokines (Barnett & Ware, 2011), and cytokine measurements in BALF of patients before and after the onset of ALI/ARDS have provided valuable insights about the complexity of the inflammatory response that occurs in the lungs (Martin, 1999). TNF‐α, IL‐6 (Wang et al., 2015), IL‐1β, TGF‐β1 (Ganter et al., 2008), CINC‐1 (Sawant et al., 2015), TIMP1 (Madtes et al., 2001), and WISP‐1 protein (Li et al., 2012) have been reported to contribute to the development of ALI. Consistent with previous reports, we found those mediators of ALI upregulated in BALF of bleomycin treated rats within 1 to 3 days after the challenge.

The proinflammatory cytokines TNF‐α, IL‐1β and IL‐6 are elevated in the plasma and BALF from patients with ALI/ARDS (Park et al., 2001). Bleomycin induced IL‐1β, TNF‐α, and IL‐6 expression on Day 3 at all investigated doses in our rat model. We also showed that 2 U/kg bleomycin increase TNF‐α and IL‐1β levels as early as on Day 1, and these cytokines remain upregulated until Day 3. TNF‐α and IL‐1β release MCP‐1, MIP‐1α, IL‐6, and lead to the production of ROS and decrease the expression of epithelial sodium (ENaC) and Na + ‐K + ‐ATPase channels (Blondonnet et al., 2016). We noticed an aberrant increase in IL‐6 on Days 2 and 3 which was greater than the increase observed on Day 1 and suggests the involvement of TNF‐α and IL‐1β in the release of IL‐6. TNF‐α plays a central role in the development of ALI by locally stimulating chemotaxis, recruitment, and activation of neutrophils. Additionally, TNF‐α induces apoptosis in alveolar epithelial lung microvascular endothelial cells and promotes edema (Lu et al., 2018). IL‐1β is a cytokine biologically active in the early phase of ALI by inducing neutrophil recruitment and activation and increasing vascular permeability via the integrin pathway (Ganter et al., 2008). We observed neutrophil recruitment and pulmonary edema at the earliest on Day 1 following bleomycin treatment suggesting it may result from the early increase of TNF‐α and IL‐1β. IL‐6 is produced by a wide range of cells including monocytes/macrophages, endothelial cells, fibroblasts, and smooth muscle cells in response to stimulation by endotoxin, IL‐1β, and TNF‐α (Tanaka et al., 2014). IL‐6 plays an important role in bleomycin‐induced lung inflammation and fibrosis, possibly through the upregulation of TGF‐β1 and MIP1α (Saito et al., 2008). In our data, we noticed an increase of IL‐6 and TGF‐β1 on Day 2 and 3, but not on Day 1, suggesting a role of IL‐6 in upregulating TGF‐β1. TGF‐β1 plays a critical role in the resolution of tissue injury and development of pulmonary fibrosis (Pittet et al., 2001), and its concentration is markedly increased in BALF as early as 24 h after the onset of ARDS (Burnham et al., 2014). With bleomycin injury, TGF‐β‐inducible genes increase as early as 2 days after the induction, a time point that precedes the maximal increase in alveolar flooding (Kaminski et al., 2000). In our study, we also saw the earliest increase of TGF‐β1 on Day 2 following bleomycin challenge which is consistent with previous reports.

CINC‐1, a rat cytokine chemoattractant, is a very potent inducer of neutrophil chemotaxis and infiltration (Koto et al., 1997). CINC‐1 increase has been reported in ALI models, and pharmacological inhibition of CINC‐1 profoundly decreases neutrophils recruitment and lung damage induced by LPS (Iwamura et al., 2002). We noticed an aberrant increase of CINC‐1 on Day 1 > Day 2 and 3 post bleomycin challenge. This suggests that the increase in neutrophil recruitment on Day 1 is initiated partly by CINC‐1, TNF‐α, and local IL‐1β release, and on Days 2 and 3 sustained by IL‐6, TGF‐β, and the other mediators that are released in a time‐dependent manner after the bleomycin challenge. Increased and persistent levels of TIMP‐1 have been reported in the bleomycin model (Madtes et al., 2001), and our results confirm these findings. WISP‐1 has been reported to induce hyperplasia and proliferation of alveolar epithelial cells accompanied by an increased expression of matrix metalloproteinases in the mouse bleomycin model (Konigshoff et al., 2009). In ventilator‐induced ALI models, WISP‐1 plays a role in modulating and/or amplifying Toll‐like receptor 4–mediated signaling (Li et al., 2012). We noticed WISP‐1 increased in our model on Day 3 > Day 2 > Day 1 which could suggest bleomycin‐initiated hyperplasia and proliferation of alveolar epithelial cells via release of WISP‐1. Collectively, several mediators implicated in ALI were elevated in our bleomycin model and correlated with increased lung inflammation and edema formation within Day 1 to 3 of administration. To better establish causality, future work should be focused on using specific inhibitors in the rat bleomycin model.

Bleomycin increases the lung collagen content in rodent on Days 7 to 21 post challenge (Peng et al., 2013; Tashiro et al., 2017). Our results showed significant collagen content on Day 3 post challenge, which suggests initiation of fibrogenesis. TGF‐β1 and Galectin‐3 are implicated in EMT, myofibroblast activation and collagen production (Fernandez & Eickelberg, 2012; Nishi et al., 2007). We showed activation of the TGF‐β1 pathway by phosphorylation of Smad2/3 and increased expression of Galectin‐3 due to bleomycin on Day 3 (Mackinnon et al., 2012). In the current study, increased expression of Vimentin and Fibronectin confirmed the activation of fibrogenesis and formation of ECM (Yin et al., 2018). Activation of the TGF‐β/Smad signaling pathway, along with increased Galectin‐3, Vimentin, and Fibronectin protein expression correlates with increased collagen content due to bleomycin. Lastly, we detected pronounced histopathological changes using H&E staining in bleomycin challenged lungs (inflammatory cell infiltration, interstitial edema, and thickness of alveolar septa) (Matute‐Bello et al., 2008).

The data described in this report show bleomycin induces characteristics of ALI in our 3‐day rat model comparable to other ALI models (Matute‐Bello et al., 2008; Matute‐Bello et al., 2011). Approximately 2 U/kg bleomycin i.t. produced intense accumulation of inflammatory cells (especially 50–60% neutrophils) in the lung, robust pulmonary edema, increased inflammatory cytokines/biomarkers network, activation of signaling pathway, biomarkers, and histopathological changes in the rat lungs within 3 days after the challenge. Biomarkers play a fundamental role in understanding the mechanisms and pathophysiology underlying lung injury and are the basis to develop future therapeutic strategies as well as to evaluate response to therapy. Our framework of BALF cytokines provides insights on possible mechanisms underlying bleomycin‐induced ALI. Our detailed characterization of ALI kinetic sheds light on contributing factors and ALI progression. Furthermore, TGF‐β1, IL‐1β, TNF‐α, IL‐6, TIMP‐1, WISP‐1, Galectin‐3, Vimentin, and Fibronectin are well documented for their roles in myofibroblast activation during fibrogenesis (Borthwick et al., 2013; Mackinnon et al., 2012; Yin et al., 2018). This suggests that our data set captures some of the early events (within 3 days) of lung injury before the development of full fibrosis in the rat bleomycin model and has implications for studying the mechanisms involved in ALI and in the development of therapeutic interventions.

## 5 CONCLUSION

To our knowledge, this is first systematic report demonstrating dose‐dependent effects of bleomycin, kinetic of ALI progression, and robust and reproducible framework of ALI readouts in a 3‐day rat model. Our data set represents robust features of experimental ALI, reveals underlying mechanism of bleomycin‐induced rat ALI and insights on the early events in the rat bleomycin fibrosis model, adding to the knowledge of current ALI models and providing preclinical data to understand mechanism of action of therapeutics under investigation.

## FUNDING INFORMATION

This study was supported by the National Institutes of Health (https://www.nih.gov/grantsfunding) through grants awarded to JES P01HL119165 and the funders had no role in study design, data collection and analysis, decision to publish, or preparation of the manuscript.

## CONFLICT OF INTEREST STATEMENT

The authors declare that they have no competing interests.

## ETHICS STATEMENT

All experimental procedures were approved by PRISM‐Institutional Animal Care and Use Committee.

## Supporting information


Figure S1.

Figure S2.

Figure S3.

Figure S4.

Figure S1–S4.

## Data Availability

All data are available in the main text or the supplementary materials.
