# Supplementary figures and images for: Characterization of acute lung injury in the bleomycin rat model
Source: Physiol Rep. 2023 Mar 10;11(5):e15618. doi: 10.14814/phy2.15618 (PMC10005890; doi:10.14814/phy2.15618)

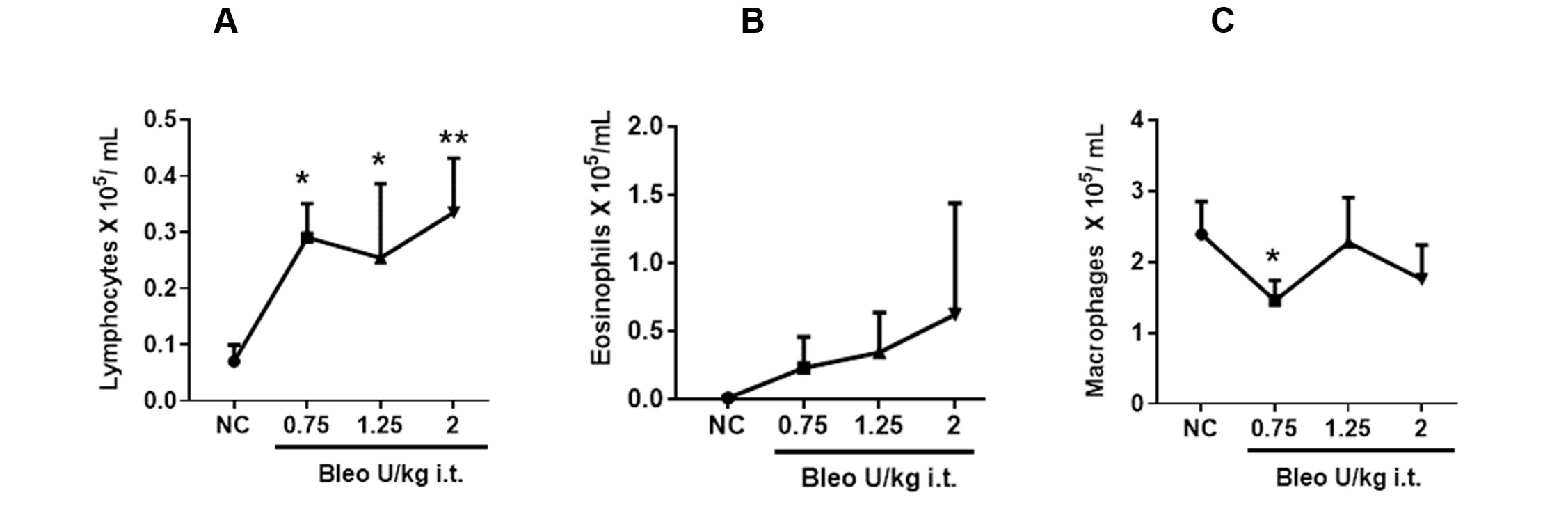

Supplement: Supplementary file 1 — Figure S1. [file PHY2-11-e15618-s003.jpg]

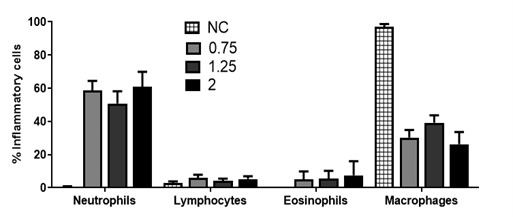

Supplement: Supplementary file 2 — Figure S2. [file PHY2-11-e15618-s001.jpg]

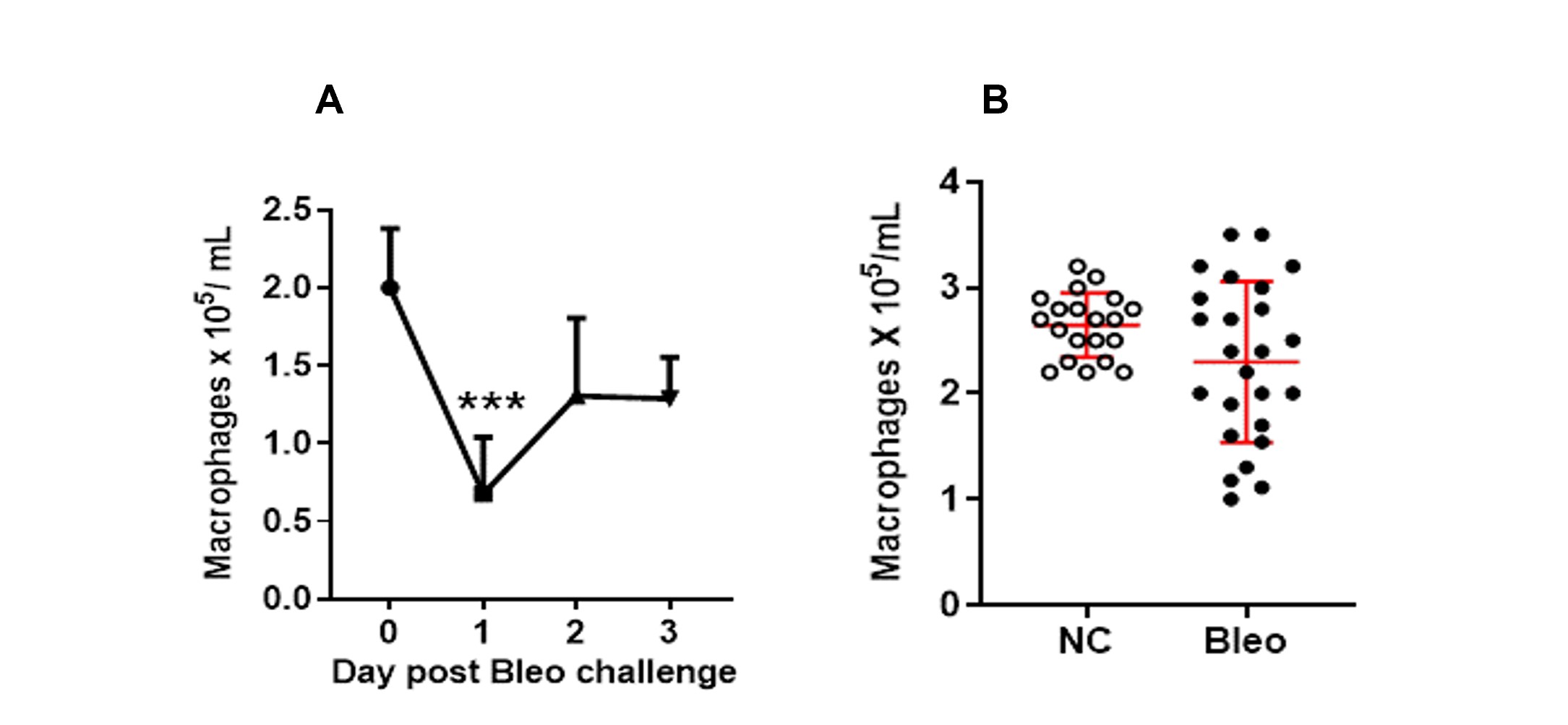

Supplement: Supplementary file 3 — Figure S3. [file PHY2-11-e15618-s005.jpg]

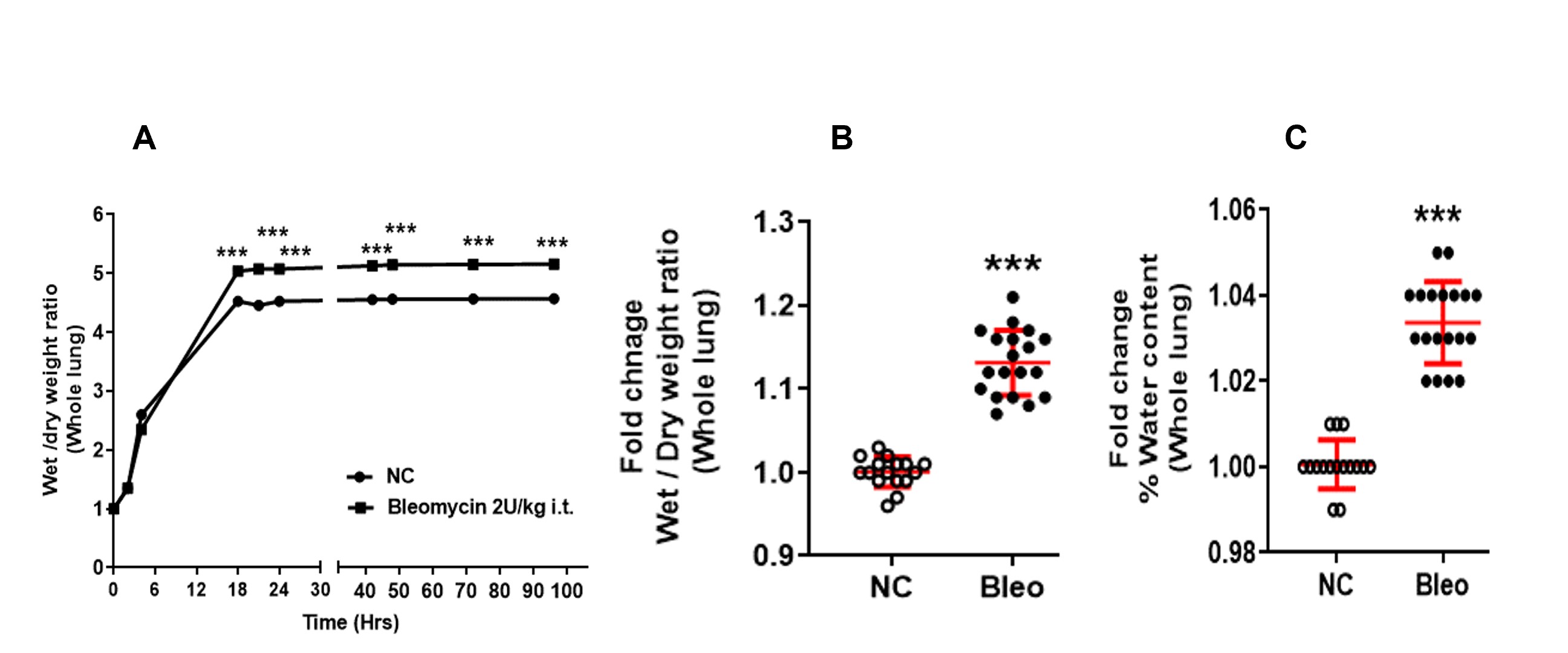

Supplement: Supplementary file 4 — Figure S4. [file PHY2-11-e15618-s004.jpg]
