# Supplementary material for: Characterization of acute lung injury in the bleomycin rat model
Source: Physiol Rep. 2023 Mar 10;11(5):e15618. doi: 10.14814/phy2.15618 (PMC10005890; doi:10.14814/phy2.15618)
Supplement: Supplementary file 5 — Figure S1–S4. [file PHY2-11-e15618-s002.docx]

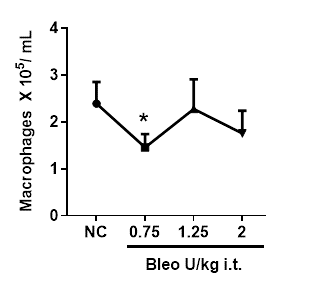

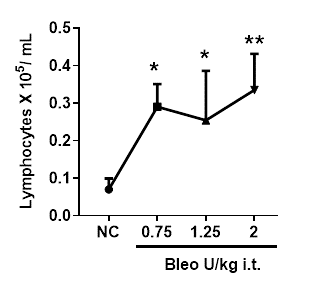

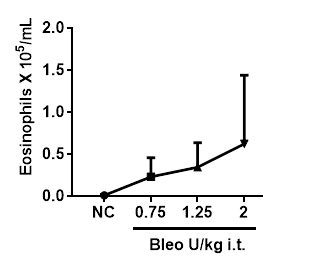
 **A B C**

**Supplemental Figure 1.** Effect of i.t. bleomycin concentrations on (A) Lymphocytes, (B) Eosinophils (E) Macrophages. Data are expressed as mean ± SD of n = 4-5 rats/group*.* **p<0.05; **p<0.01; and ***p<0.001 Vs NC.*


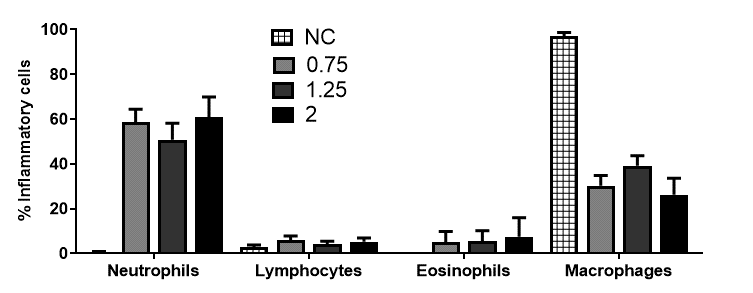


**Supplemental Figure *2.*** Effect of i.t. bleomycin concentrations on the percentage of BALF inflammatory cells. Data are expressed as mean ± SD of n = 4-5 rats/group.

**A B**


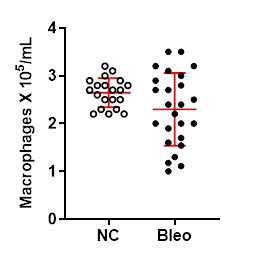

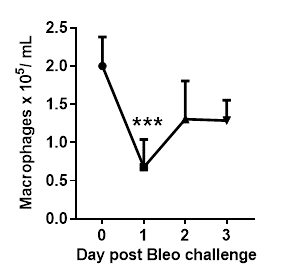


**Supplemental Figure *3.*** **Effect of bleomycin 2 U/kg i.t. challenge on kinetic of macrophages (A) and on macrophages on day 3 (B).** Data are expressed as mean ± SD of n = 5 rats/group for (A)*, n* = 20-25 rats/group for (B)

**A B C**


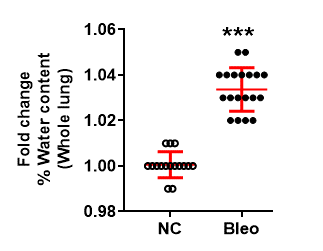

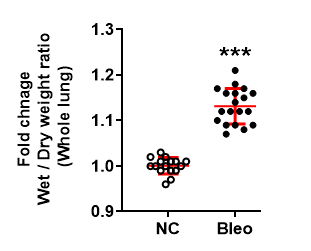

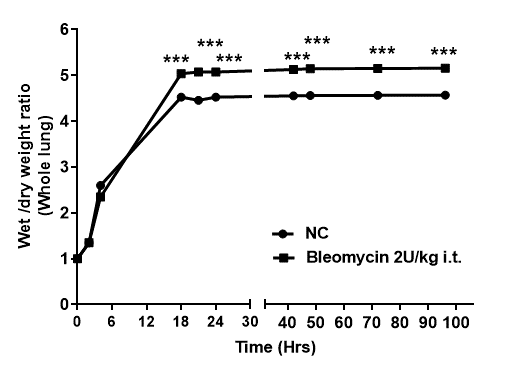


**Supplemental Figure *4.* Effect of bleomycin i.t. challenge on pulmonary edema in 3-day rat bleomycin model.** The lungs were weighed at the time of sacrifice (wet weight) and dried in an oven at 45 ^o^C. At 2, 4, 18, 21, 24, 42, 48, 72 and 96 hrs weight was measured (dry weight). (A) Change in whole lung wet to dry weight ratio over time. Data are expressed as mean ± SD of n= 2-5, (B) Fold change in wet to dry weight ratio at 24 hr, (C) Fold change in percentage water content at 24 hr. Data are expressed as mean ± SD for n = 16-19 rats/group ****p<0.001 Vs 0 hr or NC*.
